# Layer-By-Layer: The Case for 3D Bioprinting Neurons to Create Patient-Specific Epilepsy Models

**DOI:** 10.3390/ma12193218

**Published:** 2019-10-01

**Authors:** Natasha Antill-O’Brien, Justin Bourke, Cathal D. O’Connell

**Affiliations:** 1 BioFab3D, Aikenhead Centre for Medical Discovery, St Vincent’s Hospital Melbourne, Fitzroy, VIC 3065, Australia; 2 ARC Centre of Excellence for Electromaterials Science, Intelligent Polymer Research Institute, Innovation Campus, University of Wollongong, NSW 2522, Australia; 3 Department of Medicine, St Vincent’s Hospital Melbourne, University of Melbourne, Fitzroy, VIC 3065, Australia

**Keywords:** three-dimensional (3D) models, bioprinting, 3D printing, patient specific disease modelling, brain, neural network, 3D scaffolds, organoids, bioink

## Abstract

The ability to create three-dimensional (3D) models of brain tissue from patient-derived cells, would open new possibilities in studying the neuropathology of disorders such as epilepsy and schizophrenia. While organoid culture has provided impressive examples of patient-specific models, the generation of organised 3D structures remains a challenge. 3D bioprinting is a rapidly developing technology where living cells, encapsulated in suitable bioink matrices, are printed to form 3D structures. 3D bioprinting may provide the capability to organise neuronal populations in 3D, through layer-by-layer deposition, and thereby recapitulate the complexity of neural tissue. However, printing neuron cells raises particular challenges since the biomaterial environment must be of appropriate softness to allow for the neurite extension, properties which are anathema to building self-supporting 3D structures. Here, we review the topic of 3D bioprinting of neurons, including critical discussions of hardware and bio-ink formulation requirements.

## 1. Introduction: The Clinical Need for In Vitro Neural Models

Neurological disorders, such as schizophrenia, bipolar disorder, Parkinson’s disease, Alzheimer’s disease and epilepsy, affect nearly one in six people on the planet [1]. Often the cause and progression of these conditions is not well understood, and our understanding is hampered by limitations in the traditional techniques for studying the brain in situ. While the advent of functional magnetic resonance imaging (fMRI) has given neuroscientists the capability to non-invasively monitor brain activity in real time, fMRI is still a low-resolution technique, detecting relative hotspots of brain activity with accuracy on the scale of millimetres [2,3]. Additionally while higher resolution measurements of brain activity can be achieved through insertion of penetrating electrodes in animal models [4], animal models have a limited ability to recapitulate human pathologies, and treatments in animal experiments often do not concord with human clinical trials [5]. To better understand neurological disorders, and to screen potential treatments, new strategies are required.

New technologies are now emerging which enable a new way to study the brain—using miniature, ‘brain-like’ systems of neurons grown in the lab [6]. Using this “brain-on-the-bench” approach researchers can study how neurons communicate and self-organise, without having to invasively probe a living animal, or person. The development and use of human cell-based 3D models is anticipated to provide valuable insight into the pathophysiology of neurological disorders such as epilepsy. In particular, three-dimensional (3D) cellular and tissue models will hopefully shed new light on the cause and progress of such diseases, towards developing new and more effective therapies.

In an example of a ‘bottom-up’ approach, neural organoids can be generated from self-organising stem cell populations. The organoid strategy has received considerable attention; although limitations in terms of structural organisation, and reproducibility, are recognised. The complementary technology for ‘top-down’ biofabrication of 3D neural structures is a new frontier, which may be able to address some of the limitations of organoid strategies. However, the goal of 3D bioprinting neurons brings its own challenges, namely that of developing bio-ink formulations, which are simultaneously ‘printable’ and permissive to neural differentiation and neurite outgrowth.

Here, we review the topic of 3D bioprinting of neurons, with a focus on epilepsy modelling as an example application. In particular, we aim to provide critical discussions of several important questions including: Why 3D? Why 3D bioprinting? What material properties are required to achieve 3D neural networks? What are the hardware and biomaterial constraints for 3D bioprinting, and how do these conflict with requirements for 3D neuronal culture? What is the state of the art for 3D bioprinting neurons?

### 1.1. Epilepsy

Epilepsy is the fourth most prevalent neurological condition worldwide and is a debilitating and poorly understood disorder. Epileptic disorders present as uncontrollable seizures which can be dangerous to the patient. Patients may have congenital epilepsy, or it may develop throughout life [7,8]. There are a variety of epileptiform disorders with differing causes such as genetic predisposition, brain injury, stroke or tumours. However, epilepsy is idiopathic in one third of patients [7,8,9,10].

Treatment can involve dietary changes, surgical intervention or antiepileptic drugs (AEDs). AEDs can be used as monotherapies or in combination with other treatments including other AEDs [7,9,10]. Health care providers prescribe AEDs based on AEDs they have had success with in other patients, however this is an inefficient approach typically based on trial and error [11]. After cycling through various monotherapies, combinations of AEDs are tried with some patients taking several different AEDs at once. AEDs can have debilitating side effects and it can take a long time to find effective treatment, if at all. Indeed, approximately one third of patients are resistant to treatment with AEDs. 

#### 1.1.1. Limitations of Animal Models for Epilepsy

Electrode imaging in vivo only provides limited information about the brain activity of the patient; and current animal models of epilepsy have their shortcomings [11,12]. For example, one of the most widely used models of acquired temporal lobe epilepsy (TLE) is the kindling model, where electrodes with short bursts of electrical activity are applied repetitively to the limbic brain regions, often the amygdala or hippocampus, of an animal [12]. The resulting seizures, the symptoms, are very similar to those seen in clinical settings in human patients with TLE. However, the neuropathological manifestations between the human and the animal model have marked discrepancies. In humans, TLE appears as hippocampal sclerosis with damage to the amygdala; whereas kindled rats exhibit no hippocampal damage even after 300 electrical stimulations [12]. This suggests that animal models can be misleading if symptomatically they appear to mimic human disease, yet the symptoms arise from a different pathological source. Therefore, more patient specific modelling is desirable as AEDs could be tested on the patient specific neural tissue to directly observe their effect on neuron activity.

#### 1.1.2. Limitations of In Situ Methods

Electrophysiological recording of brain activity in vivo is an invasive method that is limited by feasible electrode placement and health of experimental subjects [13]. A non-invasive method of studying brain function is functional magnetic resonance imaging (fMRI). The fMRI method exploits the differences in magnetic susceptibility of oxygenated and deoxygenated hemoglobin to create an index of brain activity termed blood oxygenation level-dependent (BOLD) contrast MRI [14]. While the link between neuronal populations and cerebral hemodynamics have been established, the nature of this link is a matter of contention, and meanwhile there is dispute over the reproducibility of recordings and interpretation of the data [14]. The most common interpretation is that increases in the BOLD signal correspond to increases in neural metabolism. Additionally, BOLD signals have considerable noise from normal physiological processes such as breathing and cardiac function. BOLD signals are affected by many extrinsic factors such as amount of sleep, diet, and exercise which can confound results [14]. Additionally, the highest resolution of fMRI is 300 µm meaning that the effects of pharmacological treatment cannot be explored at the cellular level using fMRI [15]. fMRI is a valuable tool that could be used in the future in conjunction with neural in vitro models to elucidate the effects of pharmacological interventions at both a cellular level and whole organ level. 

### 1.2. Personalised Disease Modelling

#### 1.2.1. The Advent of Induced Pluripotency Stem Cells

While harvesting a patient’s neurons to create a “disease-in-a-dish” model would be invasive and unethical, recent biotechnological developments have introduced the possibility of creating patient specific disease modelling using stem cells. In 2006 Yamanaka and Takahashi pioneered a method using retroviral vectors that could reprogram terminally differentiated mature murine fibroblasts to become pluripotent stem cells. Induced pluripotent stem cells (iPSCs) can differentiate into any cell type and can be generated by the introduction of four transcription factors, Oct4, Sox2, Nanog, and Lin28, into a target somatic cell. A major advantage of this technique is that the iPSCs are genetically identical to the donor cell [16]. Therefore, tissue constructs grown from iPSCs will not be rejected by the donor’s immune system upon transplantation; and can be used to create genetically specific patient disease models [17,18,19]. Barsby et al. (2018) generated functional excitatory and inhibitory neurons from iPSCs [17].

Ma et al. (2004) demonstrated that stem cells grown in collagen gels with neural differentiation media, mimic the differentiation of central nervous system (CNS) development in vivo [20]. Via iPSC technology, patient specific disease models can be grown with the patient’s own genetic material. Subsequent epileptogenesis and screening of the tissue model with various AEDs can bypass the existing prolonged AED selection process; and possibly indicate optimal drug dose concentrations, minimizing side effects [21]. As a clinical tool personalized disease modelling of neural disorders will improve patient outcomes by facilitating appropriate AED selection on an individualized basis, accelerating the process of finding an AED regimen that results in the patient being seizure free and improving patient quality of life. If clinicians had access to a specific model of a patient’s epileptic brain tissue, they may be able to rapidly ascertain the efficacy of AEDs for that patient [21,22].

#### 1.2.2. Organoid Models

**Figure 1:**
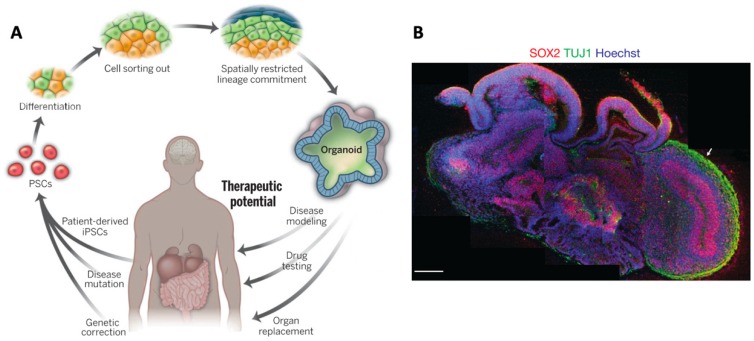
Developed from patient cells. (**A**) Organoids can be developed from patient induced pluripotent stem cells (iPSCs) for disease modelling and drug testing. Differentiated cells are self-organizing akin to organogenesis in vivo. [25]. (**B**) Cerebral organoid with heterogenous tissue regions observed via immunostaining: SOX2+ progenitor cells labelled red, TUJ1+ neurons labelled green, and nucleic acid Hoechst stained blue [27]. Images reproduced with permission from [25,27].

Organoids are simplified organs on a miniature scale (Figure 1A). Brain tissue organoids have been generated by culturing stem cells in culture conditions and differentiation media that drive the stem cells to differentiate into precursor cells that in vivo would eventually become a specific brain region. Current progress in brain organoid technology and its potential impact has been reviewed elsewhere [6,23,24,25,26]. Here, we will limit our discussion to a description of some of the main advantages and limitations of the organoid approach.

In a seminal 2013 paper, Lancaster et al. demonstrated many of these advantages including: Generation of cerebral organoids displaying discrete brain regions (Figure 1B); recapitulation of dorsal cortical organization; recapitulation of human cortical organization; as well as formation of functional cerebral cortical neurons [27]. In a major advance, the authors also demonstrated that organoids derived from a human microcephaly patient recapitulated features of the pathology. This pioneering work has been extended by many other laboratories in recent years.

For example, Jo et al. (2016) developed organoids consisting of distinct layers of neuronal cells expressing characteristic markers of human midbrain. Electrophysiological recordings demonstrated the presence of functional neurons in approximately 20% of the population; and dopamine production in 3D midbrain-like organoids (MLOs) [28]. Neuromelanin-like granules were detected in MLOs that were similar in structure to those isolated from human substantia nigra tissues. Quadrato et al. (2017) performed gene analysis in >80,000 cells from human brain organoids cultured in Matrigel over nine months, finding cells belonging to various endogenous classes including retinal and cerebral cortical cells [29]. Dendrite extension and the development of dendritic spines was observed in mature organoids and the spontaneously-active neuronal networks were confirmed by electrophysiological analysis [29]. Bagley et al. (2017) developed the fusion method where both dorsal and ventral forebrain regions were fused into one organoid body with a dorsal-ventral axis [30]. In addition to microcephaly, disease pathogenesis models have been developed for autism spectrum disorder, Sandhoff disease, Miller-Dieker syndrome, and schizophrenia [27,31,32,33,34,35,36,37]. Brain organoids have also been developed for Dengue and ZIKA viral infection [34,38,39]. While these studies illustrate the utility of organoids as neurodevelopmental disease models, they typically only illustrate the effects of chromosomal deletion on neurodevelopment without showing how the functional activity of neuron firing is affected. 

Some disease models derived from iPSCs have been developed for conditions which exhibit epileptiform activity (e.g., Rhett syndrome, Miller-Dieker syndrome), [35,40] to date no organoid model has yet been developed specifically for epilepsy; and the differences between the firing patterns of normal versus epileptic organoids is yet to be explored. 

Organoids possess many of the limitations inherent to other in vitro neural models, in that they do not mimic the 3D shape of the human embryonic brain. In vivo, the embryonic brain development is guided by the embryonic body axes which are lacking in vitro. Consistent spatial development has been achieved in organoids, however a large variability in size and tissue structure has limited the generation of disease pathogenesis models [23,24,25,31]. Organoids are restricted to a small size (maximum of several millimeters), due to the lack of nutritional supply to deeper regions [23,24,31,41]. Although agitation of the culture promotes the self-organization of neurons, a circulatory system would be necessary to allow metabolite exchange and growth factor diffusion for the organoid to develop the cortical plate and late-developing neuronal structures [30,38]. 

## 2. In Vitro Neural Models

### 2.1. 2D versus 3D Neural Culture

2D neuronal cultures have been the standard method for studying neuronal activity in vitro for several decades. The relative simplicity and versatility of the 2D set-up has allowed for many probing experiments to gain insight into the influence of substrate mechanics [42,43] or nanotopography on neuron phenotype [44]. Personalised disease models have also been developed in 2D cultures. For example, a 2D model of a childhood form of epilepsy PCDH19-Girls Clustering Epilepsy was developed by Homan et al. (2018). They generated disease model neural stem cells (NSCs) with dysfunctional PCDH19, which resulted in asynchronous neurogenesis and the loss of apical-basal polarity in disease model cells [45]. The differential timing of neuron maturation may partially contribute to network dysfunction and epileptogenesis. Patient specific disease models of Dravet syndrome, a form of infantile epilepsy, have been developed from neurons derived from iPSCs [19,46,47]. The neurons in these patient specific models displayed enlarged and persistent sodium channel activation leading to hallmark epileptic neuron electrophysical activity such as hyperexcitability, increased firing, and more intensive evoked action potentials compared to healthy patient controls [48]. Lui et al. (2013) found that the electrophysiological behaviour of the Dravet syndrome patient derived neurons, was comparable to healthy control neurons after treatment with phenytoin, a common antiepileptic drug that acts by blocking the activation of voltage gated sodium ion channels.

However, it is becoming increasingly recognised that 2D in vitro models are limited in their ability to model live tissue [49]. The brain is not a monolayer of cells; although much information can be gained from studying how neurons interact with each other in planar cultures, it is integral to include interactions occurring in 3D to recapitulate brain function.

**Figure 2:**
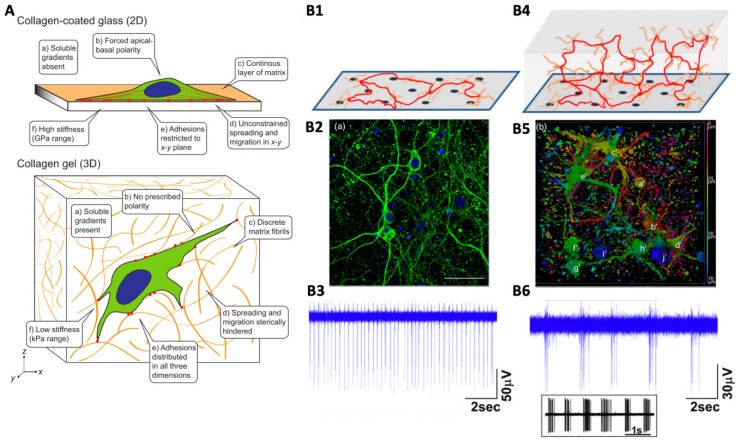
(**A**) Two-dimensional (2D) and three-dimensional (3D) present very different environments [50]. (**B**) Network formation in 2D and 3D. Bourke et al. (2018) cultured day 18 rat embryonic cortical in collagen type I 2D and 3D cultures. Schematic of 2D (**B1**) and 3D (**B4**) network formation, red indicates network activation. Immunofluoresence of 2D cultures (**B2**) and 3D cultures (**B5**) imaged at 35 days in culture. Micro electrode array (MEA) recordings from the neuronal networks of 2D **(B3)** cultures and 3D (**B6**) cultures after activation (indicated by arrows) of K-L-glutamic acid addition [58]. Inset, adapted from Magill et al. (“Copyright 2000 Society for Neuroscience”), of in vivo MEA recording from rat subthalamic nucleus similar to the 3D culture recordings [59]. Images reproduced with permission from [50,58,59].

As described in a seminal review by Baker et al. (2012) two-dimensional, monolayer culture presents an environment that is quite different from the in vivo situation (see Figure 2A ): (a) In vivo, the orientation and migration of cells is orchestrated in part by gradients of soluble factors [50,51]. In 2D culture, however, factors are typically homogeneously dispersed throughout the medium. This means an important signalling mechanism is absent. For example, soluble growth factors have been demonstrated to guide neuron migration and dendrite extension [51]. (b) The substrate is typically much stiffer than natural matrix in vivo, the flatness of the substrate forces a polarity (i.e., a one-sidedness) on the cells which is unnatural. Since the distribution of adhesion sites on the cell membrane is important for regulating cell function, these changes in cell geometry can directly impact cell function. (c) Natural extracellular matrix (ECM) is a complex environment containing arrangements of different proteins structured at the micro and nanoscale. Meanwhile, 2D culture is typically conducted on bare surfaces, or on a monolayer of a particular cell adhesion protein (e.g., collagen type 1, laminin or fibronectin). (d) In the body, the spreading of cells is constrained since the cells are ‘packed in’ by surrounding ECM. On 2D surfaces, however, the cell is unconstrained in the x-y plane, and is free to spread. Such spreading creates particular stresses in the cell body that are particular to the 2D situation. In particular, anchorage dependant cells such as neurons require adhesive interactions with neighbouring cells and ECM to recapitulate normative cell morphology in vitro [52,53]. (e) In the body, cell adhesion sites surround the cell, while in 2D these adhesion sides are restricted to the x-y plane [54,55,56]. The distribution of adhesion sites is known to impact cell function. (f) Finally, the mechanical properties of 2D substrates for cell culture are typically far higher (i.e., orders of magnitude higher) than exist in the body. Substrate stiffness is known to influence cell function, and the differentiation of stem cells in particular [42,56].

In addition to these considerations of how the microenvironment affects cell behaviour in 2D versus 3D, the dimensionality itself can have profound effects on how cells communicate. Neurons grown in 2D can only form synaptic connections with cells along one plane, meanwhile neurons grown in 3D culture can connect with surrounding neurons in all three dimensions (see Figure 2A). Lai et al. (2012) compared 2D and 3D cultures of mouse superior cervical ganglion neurons to freshly dissected ganglion tissue. Calcium flux assays demonstrated that in response to high potassium depolarization the intracellular calcium release of 3D cultured neurons was strikingly similar to that of neurons in the freshly dissected tissue; whereas 2D cultured neurons showed a significant increase in intracellular calcium release in comparison [57]. Immunostaining revealed that voltage gated calcium channel (VGCC) distribution in 2D cultured neurons were found to be tightly clustered together on the plasma membrane, whereas in 3D cultured and tissue neurons they were diffused over the plasma membrane; abnormalities in VGCC have been implicated in epilepsy (Zamponi et al., 2010), further emphasizing the need for 3D epilepsy disease models. Lai et al. (2012) demonstrated that membrane architecture and signalling behaviour are altered in 2D, but not 3D cultured neurons compared to that of native tissue.

Bourke et al. (2018) grew embryonic day 18 rat hippocampal cells in both 2D culture and as a 3D culture. The electrophysiological behaviour of neurons within both cultures was assessed via microelectrode array (MEA) at 35 days of culture (Figure 2B). Both 2D and 3D cultures were demonstrated to have glutamate sensitive neurons present with synchronous firing periods [58]. However, the 2D culture exhibited a short repetitive bursting pattern with each activation of the pace making region resulting in a single short burst at each electrode. In comparison, the 3D culture exhibited bursts of extended duration interspersed by dormant periods. The pattern of extended bursting indicated oscillatory activation through multiple network pathways of differing path lengths, demonstrating the establishment of extensive network formation throughout the collagen gel. Magill et al. (2000) reported a similar extended bursting pattern in the rat hippocampus in situ [59], therefore Bourke et al. (2018) demonstrated that neurons in 3D culture form functional networks that are not achieved in 2D. The implication is that neurons in 3D culture mimic the electrophysiological behaviour of neurons in vivo much more closely than neurons cultured in 2D, as illustrated in Figure 2B.

2D hydrogel cultures of astrocytes have also been demonstrated to yield lower populations of early astrocyte marker GFAP^+^ compared to 3D cultures, where astrocytes have a tendency to overgrow [60,61]. Cell morphologies remained round in 2D cultures compared to the stellate and perivascular-like cells observed in 3D cultures, which resemble the morphology of early astrocytes in vivo [60,62].

### 2.2. Biomaterials Requirements for 3D Neuronal Culture

To achieve 3D culture, cells are typically encapsulated in a hydrogel biomaterial which plays an early role as a rudimentary ECM [54,55,56]. The ideal biomaterial would be biomimetic of the target tissue when cultured in 3D, mimicking the mechanical, structural, biochemical, and diffusive properties of the target tissue, while remaining biocompatible with cells [50,63]. Here, we will discuss some of these requirements in turn.

#### 2.2.1. Mechanical Characteristics of the Brain

To fabricate an anisotropically accurate model of the brain in vitro presents a formidable tissue engineering challenge. The brain is arguably the most highly complex organ in higher vertebrates, and is also extremely soft, with an elastic modulus of less than 1 kPa [64]. As a structure the brain is not stiff enough to support its own weight under gravity; being supported by cerebrospinal fluid in situ [64]. Cell density also plays a role in the stiffness of the brain; as cells proliferate the stiffness and amount of ECM components secreted by the cells increases [56,65]. The influence of the mechanical environment on neural differentiation has been observed in vitro, with softer environments <500 Pa promoting the differentiation of adult neural stem cells (NSCs) towards a neuronal lineage, whilst stiffer substrates (1000–10,000 Pa) promote glial differentiation [42].

#### 2.2.2. ECM Composition of the Brain

The development and differentiation of precursor neural cells in vitro is a recapitulation of the development on the brain in utero. Thus, in vitro constructs aim to mimic the foetal brain microenvironment. Sood et al. (2016) characterized foetal and adult porcine brain ECM and found that foetal ECM was comprised of 417.8 µg/mg collagen and sGAG 406.6 µg/mg, whilst adult brain ECM had a pointedly higher sGAG to collagen ratio, with less collagen at 200.9 µg/mg, and sGAG concentration of 397 µg/mg [54]. Sood et al. (2016) demonstrated via protein gel electrophoresis that the collagen in both adult and foetal ECM was predominantly collagen type 1 [54]. Yet it is still largely unknown what the function of the collagens; and other fibrous ECM components such as fibronectin and vitronectin; are in the brain [66,67]. Certainly, different integrin binding sites play an important role in multiple signalling pathways [68,69]. Foetal brain tissue is markedly more gelatinous and less viscoelastic than adult brain tissue [70]. The replacement of foetal ECM proteins with homologous adult ECM proteins confers a stiffer and more robust nature to the adult brain [71]. 

This native composition suggests hydrogels as suitable materials for formulating in vitro neural culture, in particular collagen type I and hyaluronic acid.

#### 2.2.3. Microfluidic Devices: Drug Screening and Disease Modelling

Microfluidic devices, also called ‘organ-chips’ or ‘lab-on-a-chip’, consist of several microchannels with volumes of microliters or picolitres, and can be fabricated with interconnected compartments containing 2D or 3D neural culture environments. Designed compartments allow individual cell analysis of cell secretions, protein, and gene expression. Channels can enable fluid flow that can introduce gradients and model the dynamic in vivo environment [72,73]. Microfluidic devices have been used for drug screening applications and in-depth study of cell behaviour in varying disease or injured states with high throughput capability [74]. Meanwhile embedded MEAs can facilitate the study of neural network dynamics, signalling behaviour and fluid perfusion [73,75,76,77,78]. A few examples are mentioned here, and Osaki et al. (2018) provides a detailed discussion of microfluidic disease modelling of the CNS [79].

Wevers et al. (2016) used the commercially available OrganoPlate^®^ to culture human iPSC derived neural progenitor cells (NPCs), glial cells, and neurons embedded in Matrigel. OrganoPlate^®^ consists of 96 tissue ‘chips’, which have four wells that feed via capillary action into channels: A gel inlet well, medium inlet well, medium outlet well, and an observation window. Functional neurons formed with network formation was assessed by Ca^2+^ imaging. The neurotoxins 2,5 hexanedione, endosufan and methylmercury were added to media in varying concentrations, and their effects on cell viability were assessed, however the effect on neurite outgrowth was only reported for methylmercury [80]. The 96 chips allow for high throughput testing of various compounds, which could be applied to patient derived iPSCs to allow personalized drug screening.

Microfluidic devices have also been used for ex vivo study. Hippocampal slice cultures spontaneously develop seizures within a week after dissection and are used as in vitro models of post-traumatic epilepsy [81]. Liu et al. (2019) developed a six-well microfluidic device with incorporated MEAs to obtain electrophysiological recordings from six hippocampal slices simultaneously, reducing the variability between controls and experimental replicates in drug screening assays. Twelve small molecules of receptor tyrosine kinases were screened using this platform. First, the drugs were screened by biomarker assays for lactate and lactate dehydrogenase, which have been associated with seizures and seizure induced cell death; raised levels were found with several of the tested drugs. MEA recordings showed only two drugs, EGFR/ErbB-2 inhibitor and cFMS inhibitor, to have significant reduction in seizure-like electrical activity [81]. While potentially useful for drug screening, animal derived ex vivo models have some of the same limitations as animal models, in addition to difficulties in maintaining long term culture. 

## 3. Biomaterials for 3D Neuronal Culture Models

Here, we survey research into developing hydrogels for 3D culture of neurons. Hydrogels consist of hydrophilic polymer chains that crosslink to form a network. Hydrogels are an attractive material for culturing cells in 3D due to their biocompatibility, high water content, and tunable physical and chemical properties. Mechanical stiffness and pore size are tunable in many hydrogels to match hydrogel properties with the in vivo cellular environment [82,83]. In the case of neurons, the pore structure of the hydrogel must be able to support neural cell bodies, which are 10–50 µm, [83] while allowing the process of neurite extension [84]. Interconnected pores facilitate mass transport of cellular nutrients and metabolic waste [83].

Typically in the cell encapsulation process, cells are mixed with hydrogel precursors, which are then crosslinked via chemical means or changes in pH or temperature, with cells being encapsulated during the gelation process [85]. Neuronal survival is affected by many factors such as cell–cell interaction, soluble survival and growth factors, and signals from the ECM [84,86]. Natural hydrogels such as collagen often contain naturally occurring ECM signals, whilst other hydrogels require additives containing ECM signals to allow neuron survival [84]. The mechanical characteristics of the gel, i.e., stiffness and porosity, and ability to support neural growth and network formation are important factors for choosing which biomaterials may be useful for neural cultures. The assessment of electrophysiological activity to establish functionally active neurons and neural networks is critical to attain functional outcomes from biomaterial-based neural cultures, however many studies focus primarily on cell viability and morphology (Table 1). Electrical stimulation has been demonstrated to trigger or accelerate the differentiation of stem cells towards neural lineages [87,88,89,90,91], however we limit the scope of our discussion to biomaterial culture without such exogenous stimuli.

### 3.1. Collagen Based Materials

**Figure 3:**
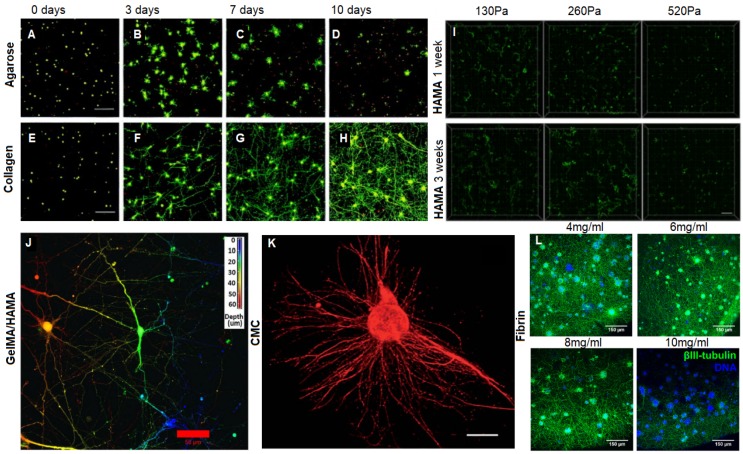
Biomaterials for 3D neural culture. (**A**–**D**) LIVE/DEAD staining of cortical neurons encapsulated in 0.5% *w*/*v* agarose gels over 10 days, scale bar 100 µm. (**E**–**H**) LIVE/DEAD staining of cortical neurons encapsulated in 0.04% *w*/*v* collagen gels over 10 days scale bar 100 µm; compared to agarose collagen is more supportive of neuron survival and maturation [84]. (**I**) Neural stem cells (NSCs) derived from iPSCs encapsulated in hyaluronic acid methacryloyl (HAMA) 1% *w*/*v* exposed to crosslinking ultra-violet (UV) light for 60, 90, and 120 s, scale bar 200 µm. Increased duration of UV exposure increases the stiffness of the resulting gel, neuron differentiation was promoted in softer gels of 130 Pa [40]. (**J**) Confocal depth decoded image of embryonic hindbrain cells encapsulated in 3.5%/0.5% *w*/*v* gelatin methacryloyl (GelMA)/HAMA at day 15. Colour coding indicates the depth of different planes along the *z* axis, scale bar 50 µm [109]. (**K**) Dorsal root ganglion (DRG) with neurite extension in carboxymethyl chitosan (CMC), scale bar 500 µm [111]. (**L**) NPCs encapsulated in varying concentration of fibrin gel after 14 days. Immunostaining for neuronal processes (β-tubulin III+) shown in green, 4′,6-diamidino-2-phenylindole (DAPI) staining of nuclei in blue, scale bar 150 µm. Neural network formation was promoted in lower concentration fibrin gels [114]. Images reproduced with permission from [40,84,109,111,114].

Collagen type I, hereafter referred to as collagen, is extracted from animal sources and stabilized in liquid form in acetic acid, it can then be crosslinked through a pH change or with chemical crosslinking agents such as genipin [58,92]. The mechanical properties of collagen are tunable via the manipulation of crosslinking agents and collagen concentration. Neurite growth has been observed to be more pronounced in collagen gels of lower concentration [20,58,93] (Figure 3E–H). The pore size of 3D collagen hydrogels decreases with increases in collagen concentration; and neurite extension tends to increase with larger pore size [94]. 3D bulk collagen gels have been shown to support neuron growth for over a month with functional neural network formation [58]. Functional network formation requires interconnecting extensive neurite extension, which was achieved in very low collagen concentration, 0.04% [58]; confirmed via MEA. Extensive interconnecting networks formed due to the very soft nature of the gel; this concentration was found to have a complex dynamic modulus of 10 Pa at this concentration [95]. 

As described above, collagen I is a native component of brain ECM in vivo, and provides binding sites encouraging neurite outgrowth such as RGD and target sequences of matrix metalloproteinase (MMP) [95]. The protein laminin is an ECM component that is often added to hydrogels to promote neuron adhesion, however the incorporation of laminin to 3D collagen gels has been demonstrated to (a) not affect the mechanical stiffness and (b) limit neurite extension in contrast to collagen only 3D gels; whereas laminin addition improves neurite extension in 2D collagen cultures [95]. Neurite growth has been observed to be more pronounced in collagen gels alone in comparison with collagen gels combined with either laminin or fibronectin [96].

### 3.2. Hyaluronic Acid Based Materials

Hyaluronic acid (HA) is a hydrophilic nonimmunogenic long chain polysaccharide found in the ECM of connective tissue; it is also integral to the structure of the CNS [54,97]. The foetal brain is rich in HA, where HA synthesis is upregulated along NPC migratory routes [40]. HA itself has a role in inflammation regulation: Low molecular weight HA is pro-inflammatory and high MW HA is anti-inflammatory [97]. High MW HA has been demonstrated to limit glial scarring after spinal cord injury or brain damage [98]. Many cell types including NPCs express the HA receptor CD44 acting as a mechano-transduction sensor; the addition of ECM motifs is often used to encourage cell development [40,99,100]. 

HA concentration can be altered to create bioinks with tunable mechanical characteristics without changing the pore size of the scaffold [13,101]. In its native form HA is rapidly biodegradable in vivo by cell secreted hyaluronidase [97]. For use in 3D modelling HA is often covalently crosslinked to form an insoluble hydrogel to increase its stability as a biomaterial scaffold [99,102]. 

One of the most popular techniques to covalently crosslink hyaluronic acid is to functionalise the polymer chains with methacrylate groups, which can be photocrosslinked through a photo-induced free-radical polymerisation reaction. Higher degrees of functionalisation result in stiffer gels and slower degradation via hyaluronidase [102]. The duration of photo crosslinking can determine the stiffness allowing another method of manipulation of the mechanical properties of methacrylated hyaluronic acid (HAMA) [103].

Softer HAMA 3D cultures drive NPCs towards a neural phenotype, whereas HAMA 3D cultures with stiffer mechanical properties akin to an adult brain favoured NPC differentiation into astrocytes [102,103]. Functionally active GABA and glutamate responsive neurons were established in 3D HAMA hydrogels supplemented with RGD, YIGSR, and IKVAV [40] (Figure 3I).

Another strategy to achieve covalent crosslinking is thiolated HA (HA-SH), which has been used to synthesise gels with storage modulus comparable to native brain tissue at 188 ± 42 Pa [102]. However at 70 days neurons were not functionally mature with cells displaying embryonic electrophysiological behaviour. HA has also been enzymatically crosslinked via the transglutimase activity of FXIIIa (Ha-TG) to create a 3D neural culture from rat embryonic cortical cells. The storage modulus of 100 Pa was soft enough to allow neurite extension with cell dendritic spines emerging after Day 5 [104]. Long term coordinated and spiking calcium activity was observed in multiple cells, suggesting functional neural network formation with glutamate responsive neurons. 

### 3.3. Other Hydrogels

#### 3.3.1. GelMA

Gelatin is hydrolyzed collagen and therefore shares many of the same ECM peptide motifs as collagen such as RGD and MMP sequences promoting cell adhesion. Gelatin methacryloyl (GelMA) is a hydrogel which covalently crosslinks via photoinitiated radical polymerization. GelMA is relatively tunable; both pore size and compressive modulus can be modulated by varying the degree of methacryloyl substitution [105]. However, GelMA cannot form stable, contiguous gels below a concentration of about 5% *w*/*v*, and so achieves compressive moduli on the order of 1000 Pa at its softest [106]. PC12 neuronal cells and NPCs encapsulated in GelMA have healed mouse brain injury and lead to significant functional recovery in murine spinal cord injury (SCI), respectively [107,108]. Neurons from the gigantocellular nucleus have been encapsulated in 3D hydrogel combination of GelMA/HAMA both with and without the addition of laminin exhibited dendritic extensions, expressed β tubulin III confirming neural phenotype, and three-dimensional distribution throughout the hydrogel with network formation [109] (Figure 3J). 

#### 3.3.2. Agarose

Agarose is a non-toxic polysaccharide derived from seaweed that has been utilized as a material for 3D neural cultures. However, agarose networks are lacking in suitable cell attachment sites, and are not suitable for neuron adherence and survival [84] (Figure 3A–D) so agarose based neural cultures require supplementation with ECM proteins. Modification of agarose with covalently bound laminin via the bifunctional crosslinking agent 1′ 1 carbonyidiimidazole improved neurite extension in PC12 and dorsal root ganglion (DRG) cells [110].

#### 3.3.3. Chitosan

Chitosan is a polysaccharide derived from the exoskeletons of shellfish. Carboxymethyl chitosan (CMC) functionalized with methacrylate groups can reach compressive moduli in the range of 10–400 Pa, comparable to brain tissue, depending on concentration [64,111]. 0.5% chitosan 3D gels supported neurite outgrowth of encapsulated DRGs and hippocampal neurons. Chitosan is positively charged which promotes cell attachment and differentiation, especially into glial cells, even in the absence of ECM cues [111] (Figure 3K).

#### 3.3.4. Alginate

Algae derived alginates can be ionically crosslinked via the addition of a divalent cation such as calcium to form a hydrogel network. Palazzo et al. (2015) developed alginate hydrogels with storage moduli ranging from 10–4000 Pa, depending upon the alginate concentration and the concentration of the calcium crosslinker added. The lowest concentration of alginate, 0.1% had a storage modulus of 10 Pa with 83% of encapsulated rat primary cortical neurons viable after three days [112]. Calcium flux showed spontaneous activity in multiple cells with synchronized bursting indicative of functional neural network formation [112], however without challenging the cells with signal blockers it cannot be confirmed that the calcium spiking activity was from neural electrical activity alone. Interestingly, 0.2% alginate hydrogels had a storage modulus of 100 Pa with lower viability and less pronounced firing patterns compared to the softer 0.1% gel. This study illustrates that the softer the 3D matrix is the more amenable it is to the formation of neural networks, despite the lack of ECM integrins [112]. This study also highlights the differences between 2D and 3D culture, in 2D cultures 10 Pa environments decrease neural maturation [42]. 

#### 3.3.5. Matrigel

Matrigel is a viscous protein-rich secretion from Engelbreth-Holm-Swarm (EHS) mouse sarcoma cells that contains many ECM components [113]. Irons et al. (2008) co-cultured embryonic cortical neurons with astrocytes to create a 3D neural model using commercially available Matrigel [61]. Extensive neurite outgrowth was observed and patch clamp recordings evidenced neuron functionality [61]. However, Matrigel is known to suffer from batch to batch variability, so 3D cultures consisting of Matrigel may lack reproducibility [113].

#### 3.3.6. Fibrin

Fibrin hydrogels are produced from fibrinogen, which is derived from plasma containing Factor XIII, and crosslinks via the enzymatic activity of thrombin. Increased fibrinogen concentration results in gels of higher stiffness and decreased pore size, fibrinogen concentration 4 mg/mL, 6 mg/mL, 8 mg/mL and 10 mg/mL resulted in pore sizes of 0.71 µm, 0.65 µm, 0.53 µm and 0.49 µm; and elastic moduli of 400 Pa, 700 Pa, 1000 Pa and 1200 Pa [114]. Bento et al. (2017) established 3D fibrin hydrogels embedded with neural stem/progenitor cells (NSPCs) derived from mouse embryonic stem (ES) cells [114]. A range of fibrinogen concentrations were explored, with elastic moduli from 400–1200 Pa. While fibrinogen concentration did not significantly affect cell viability, lower concentrations formed dense extensive neural networks (Figure 3L). After 14 days NSPCs were secreting ECM proteins laminin, fibronectin, and collagen type IV, and actively remodelling the microstructure of the fibrin gel using matrix metalloproteinases (MMP) and matriptase [114]. 

#### 3.3.7. Poly(ethylene glycol) (PEG)

Synthetic poly(ethylene glycol) (PEG) hydrogels have been used to develop multicomponent neural constructs that mimic brain tissue [18]. Thiol-ene photopolymerization was utilized to crosslink 8-arm PEG-norbornene molecules with RGD and MMP-degradable peptides to promote cell adhesion. Neural progenitor (NP), endothelial and microglial cells derived from iPSCs were added to the culture at different times to mimic brain development in vivo. Genetic analysis showed that NPs in multicomponent cultures had more uniform gene expression compared to NP only constructs; this method is an alternative to organoid generation as the cellular organisation and gene expression profiles were highly reproducible [18].

### 3.4. Disease Modelling in Bulk Gels

3D hydrogel cultures have been developed as brain disease models, using patient derived iPSCs or via manipulation of culture conditions. Although bulk gel 3D cultures lack the microarchitecture of native tissue, valuable insights have been gained via bulk gel disease models. High molecular weight HAMA has been utilized to develop 3D models of glial scar formation, with an elastic modulus similar to that of native brain tissue of 200 Pa [13]. Neural behaviour is often the focus in epilepsy research, however the formation of glial scars in response to trauma is thought to be a leading cause of acquired epilepsy [119]. Surgical removal of glial scars has alleviated seizures in cases of drug-resistant epilepsy [119] suggesting that the role of glial scars in epileptogenesis is an avenue for further study.

To explore neural migration in Rhett syndrome, a symptom of which is epileptic seizures, patient derived iPSCs were compared to nonaffected parental control derived iPSCs. Rhett syndrome is attributed to mutations in the gene encoding methyl-CpG-binding protein-2 (MeCP2) [40]. Patient derived NPCs only migrated 70% compared to the migration of parental control NPCs. Patient derived neurons exhibited decreased neurite outgrowth and less synaptic connections compared to controls [40]. When MeCP2 function was restored in patient derived cells the migratory behaviour of NPCs was rescued, indicating that inhibited neural migration due to defective MeCP2 during early neurodevelopment contribute to the pathology of Rhett syndrome [40]. 

Approximately 5% of people hospitalized for traumatic brain injury (TBI) develop post-traumatic epilepsy (PTE) [120]. Al Rifai et al. (2015) implanted PC12 neural cells encapsulated within a 4% and 8% GelMA into mice that had sustained TBI; one week after implantation the injury had disappeared [107]. In vitro after three days axon like projections were observed; after seven days the implanted constructs stained positive for PC12 markers and were found to be non-immunogenic and non-inflammatory [107].

In a model for the pathogenesis of Alzheimer’s disease, 3D collagen matrix with embedded β-amyloid aggregates was seeded with PC12 cells, no toxicity was observed when cells were separated from β-amyloid aggregates [94]. Only upon addition of collagenase did neurites extend into the gel, neurites within 1 µm of β-amyloid aggregates exhibited stopping or turning behaviour. When cell bodies were cultured close to β-amyloid aggregates there was low cell viability and no neurite extension [94], these results demonstrate that the toxicity of β-amyloid aggregates is dependent upon the proximity to neurons. In another β-amyloid study human iPSC derived NPCs were embedded in the commercial 3D hydrogel PuraMatrix, a self-assembling RADA-16 peptide that crosslinks upon exposure to cations [74,121]. Treatment with β-amyloid peptides was found to significantly lower NPC expression of proteins for actin stabilization and mechanotransduction pathways, drebrin and p21 activated kinases (PAK), respectively. In untreated NPCs, activated PAK and drebrin were localized in the nucleus and cytosol, and NPCs treated with β-amyloid peptides caused activated PAK and drebrin to be sequestered to submembraneous regions [121,122]. 

Choi et al. (2014) used retroviral vectors to engineer NPCs that overexpressed familial Alzheimer’s disease mutations to produce high levels of β-amyloid peptides. Engineered NPCs were embedded in 3D Matrigel, and high levels of β-amyloid and hyper-phosphorylated tau accumulated in six weeks, compared to 2D cultures where β-amyloid peptides diffused into culture media. Application of β and γ secretase inhibitors significantly lowered β-amyloid levels and also tau, demonstrating that high levels of β-amyloid peptide contribute directly to tau pathology [123]. This link had not been shown with animal models or 2D models [74,123], demonstrating the advantages of in vitro 3D disease modelling.

## 4. Strategies for Biofabricating Neural Models

Layered neural models of neurons encapsulated in bulk gels have provided insights on neural migration and neurite extension. Although 3D constructs can be created by simply pouring a bulk gel into a cast with high fidelity to the desired form, referred to as cast moulding [124], cell placement within the gel is random, and cells tend to settle at the base of the mould prior to gelation or clump together. Larger 3D bulk gels show necrosis at the core due to limited diffusion [61]. Other methods of neural biofabrication include microfluidic technology and using 3D scaffolds. Many different types of 3D scaffolds have been developed for 3D neural culture; materials vary greatly including hydrogels, glass, and polymers. Most microfluidic devices use 2D cell culture, however some 3D models have been developed for drug screening and disease modelling, discussed later in the disease modelling section. 

### 4.1. Layered 3D Neural Models

Layered 3D neural models are formed by crosslinking one layer of a bulk gel prior to deposition of the next layer. These structures are typically created through manual pipetting, and so represent a link between the bulk 3D gels already discussed, and the more robotically controlled bioprinters to follow. Layered models have demonstrated that cell seeding onto 3D gels is inferior to cell encapsulation in 3D gels in recapitulating in vivo neural morphology [118]. Layered models have been used to demonstrate that neurite extension is increased towards NGF in 3D [110]. A layered model demonstrated that NPCs migrate toward astrocytes in 3D and showed defective cell migration in patient derived MeCP2 mutant cells as a disease model for Rhett syndrome [40].

### 4.2. Gradients in 3D Bulk Hydrogels

Durotactic gradients, gradients of increasing stiffness, were demonstrated to promote neurite extension compared to gels of uniform mechanical properties [117]. Similarly haptotactic gradients, gradients of bioactive molecules, of YIGSR and IKVAV were demonstrated to promote neurite outgrowth in a synergistic manner in 3D [51]; in agreement with findings in 2D cultures [125,126].

### 4.3. 3D Scaffolds

3D scaffolds serve as a rudimentary structure to guide cell growth via topographical cues; cells are seeded onto pre-formed scaffolds. A myriad of techniques has been developed for neural scaffolds with applications ranging from disease modelling to therapeutic implants. Herein, we describe neural scaffolds with a focus on guided neurite growth, mechanical and electrophysiological properties. Colloidal scaffolds consist of layers of microbeads; silk scaffolds have served as moulds for bulk hydrogels; methods to create macroporous scaffolds have been developed and electrospinning has been used to guide neurite extension.

#### 4.3.1. Colloidal 3D Scaffolds

Colloidal scaffolds consist of layered microbead bead arrays, beads of silica [127], glass [128], and chitosan [129] have been used in the fabrication of 3D scaffolds. In such colloidal substrates, neural processes spread along and between the beads and network activation through different layers has been demonstrated with calcium flux assays [127] and MEA recordings [128,129]. Neurites, however, can only extend along bead surfaces, limiting cell–cell interaction and network activation between layers, with diminished connectivity in the z direction between layers slowing response to evoked potential [128]. Burst amplitude between the 2D control and 3D scaffold recordings were of a similar amplitude, the burst duration was longer with an often-delayed response for the 3D recordings [128]. Stimulation from electrodes at the top of the scaffold had delays of 500 ms due to the transmission down the neurons of the different layers to the bottom read-out layer in contact with the MEA [128], indicating less effective connectivity in the z direction. Tedesco et al. (2017) found similar results but with increased random spiking in neural cultures seeded within 3D colloidal hydrogel scaffolds composed of chitosan microbeads, and detected the synaptic vesicle protein synapsin at day 25 [129]. Neurites penetrated soft chitosan microbeads to a limited extent, whereas neurite extension was only upon the surfaces of glass or silica microbeads [127,128,129]. Microbeads of 0.1% and 0.2% chitosan scaffolds, were ~0.08 kPa–1.3 kPa and 15 kPa–25 kPa(elastic modulus), respectively; the softer scaffold showed more extensive network formation and functionality, demonstrating the importance of mimicking the brains in vivo mechanical environment [56,64,129]. However, the electrophysiological results of neuronal network formation in a bulk hydrogel collagen more closely resembled that observed in living brain tissue [58,59].

#### 4.3.2. Silk Scaffolds

The silk from silkworm cocoons can form a sponge like material with pore size ~500 µm and is stiff enough to be self-supporting. Tang-Schomer et al. (2014) developed 3D silk scaffolds with compartmentalized layered architectures as a cerebral model: Axon only ‘white matter’ in the centre and neurons in the outer layers as ‘grey matter’. [130]. Collagen hydrogel infusion followed seeding with primary cortical neurons. Another study found improved cell viability, network density, axon outgrowth, and accelerated functional neural maturation of 3D silk scaffold collagen composite constructs in the presence of adult decellularized ECM. These effects were enhanced further by the addition of foetal decellularized ECM and astrocyte derived matricellular proteins [54].

#### 4.3.3. Macroporous Scaffolds

Macropores are large pores acting as interconnected channels to facilitate mass transport and cell penetration throughout a 3D construct. Macropores can be fabricated using a pore creating agent, called a porogen [83,131]. For example, gaseous salt leaching involves combining a viscous polymer solution with salt crystals as a porogen in a mould; and evaporating the liquid, leaching out the salt, resulting in a hard-porous structure. Salt concentration and crystal size determines the porosity of the resulting scaffold [131]. Neurons have been shown to be viable but did not proliferate within a gas leached scaffold, probably due to the rigidity of the scaffold [57]. Nonetheless this method demonstrated the exaggerated responses of neurons cultured in 2D in calcium flux assays, compared to the high degree of similarity between 3D cultures and freshly dissected tissue [57].

Cryogels are macroporous hydrogels where crosslinking occurs at subzero temperatures, ice crystals act as porogens, disrupting the crosslinking of the hydrogels, and once thawed leave macropores within the interconnecting micropores of the hydrogel [132]. Due to the macroporous nature of cryogels they reversibly collapse under shear force, allowing them to recover their original crosslinked 3D shape after being passed through a syringe [133], referred to as shape memory. Alginate cryogels have been demonstrated to have compressive modulus of 4 kPa compared to a bulk alginate hydrogel of 42 kPA in the same concentration of alginate and same 3D shape [133]. Neural networks and cell morphology of differentiated neurons were preserved even after compression and extrusion through a needle [134]. Jurga et al. (2016) developed a 3D gelatin laminin cryogel seeded with stem cells from human umbilical cord blood cells (CBSCs); which when transplanted into rat brains were found not to induce inflammation or microglial activation, and prevented astrocytic scar formation [135]. Pore sizes varied from 20 to 160 µm, with pores < 100 µm required for cell–cell contact. CBSCs expressed MAP2, Nestin, and GFAP in medium sized pores, demonstrating their differentiation into neuronal and glial cell types due to good morphogen penetration. In smaller pores the stem cells remained undifferentiated, demonstrating the importance of pore size optimization for stem cell differentiation in 3D scaffolds [135].

A major limitation with such macroporous gels is that the low temperature or high salt concentrations, critical to the construction technique, are damaging to cells, so cells can only be added after the gel is fully formed, limiting cell encapsulation as a seeding method.

#### 4.3.4. Electrospun Scaffolds

Electrospinning is a technique used to create fine fibres with nano- or micrometer diameters. The technique involves using electric force to draw charged jets of polymer which are collected on a grounded collector; stationary collectors result in randomly oriented fibres while rapidly rotating collectors produce aligned fibres [136]. Electrospun fibres have been demonstrated to guide neurite extension and increase cell to cell contacts with fibres of polycaprolactone (PCL) and polylactic acid (PLA) [44,137]; aligned fibres were shown to increase cell viability and long term survival in a planar model of the synaptic neuromuscular junction. Neurite extension was doubled in 3D hydrogel HA-SH scaffolds with embedded PCL electrospun fibres and neurite alignment increased 66% when PCL was functionalized with gelatin or laminin [138]. Engelbreth-Holm-Swarm (EHS) ECM is derived from murine sarcoma secretions containing many cell attachment motifs [113]. Neurite length and fibre tracking were not significantly improved with functionalized PCL electrospun fibres embedded in EHS ECM [138], suggesting that hydrogels rich in ECM proteins may not be suitable for constructs where neurite extension is directed by fibres.

Standard electrospinning techniques using a flat static collector or coaxial spinning mandrel result in a 2D matt of fibres [139,140]. Often electrospun fibres are densely packed, preventing cell infiltration. Blakeney et al. (2011) developed a method of creating electrospun 3D cotton ball like structures with low density fibres, using concave non-conductive spinning plates embedded with metal needles to collect the fibres [139]. Jakobsson et al. (2017) used this method to electrospin a 3D PCL structure which was seeded with NPCs; interspersed glial (GFAP+), and neurons (MAP2+), with inhibitory (psd-95+) and excitory (gephyrin+) synaptic proteins and extensive neurite formation were observed in the lower 100 µm of the 600 µm scaffold [140]. Single channel extracellular electrode recording demonstrated spontaneous action potentials from some locations, yet other locations were silent, to confirm functional network activity MEA recordings would be required [140].

Melt electrowriting (MEW) is a technique where polymer melts are directly written in a programable path, using a layer by layer technique MEW can be used to fabricate 3D electrospun structures [141]. MEW is more reproducible than typical electrospinning which can often have chaotic fibre deposition. Schaefer et al. (2019) developed a 3D prototype neural model of MEW PCL seeded with fibroblast embedded Matrigel, and demonstrated that the MEW PCL did not interfere with whole cell patch clamp recordings [142]. Neurons are more vulnerable to environmental stressors than fibroblasts, in 2D neuron electrophysiological behaviour was found not be affected when seeded on PCL electrospun fibres [44].

By combining two electrospun polymer inputs, HAMA and HA and/or zero and 3 mM RGD, in different ratios over the course of electrospinning, durotactic, and haptotactic gradients were achieved through the 3D scaffold [92]. Cells from chick aortic arch explants infiltrated scaffolds to a greater degree towards higher concentrations of RGD and lower mechanical stiffness; this method could be applied to guide neurite extension and migration. 

### 4.4. 3D Printed Scaffolds

3D printing, also called additive manufacturing, creates 3D objects from a digital model by depositing or patterning material via a layer by layer approach. 3D printing, in this section, refers to applications where cells are seeded on a 3D scaffold after the printing process. A wide range of materials can be 3D printed, including hydrogels [143,144,145]. By creating the digital model from 3D medical images, complex anatomically correct 3D objects can be printed [146,147,148]. Computer aided design (CAD) allows easy modification of designs to optimise scaffold properties [149], such as creating channels or artificial macropores, which can be varied between prints to determine the optimal sizing for neural interconnections [143,144]. The most common 3D printing techniques used to create neural scaffolds for neural tissue engineering include photopatterning, direct laser writing, extrusion printing, and inkjet printing.

#### 4.4.1. 3D Photopatterning

3D photopatterning refers to 3D printing techniques where light is used to draw a crosslinked pattern in a photocurable material or resin. Such techniques include stereolithography, which crosslinks in a point by point fashion until each layer is completed; digital light projection, which illuminates each layer, plane by plane [105], as well as other variations [144,150].

Photopatterning based single neuron capture in 3D GelMA microgel rings has been developed as a way to model autapse formation [150]. A projection printing technique has been used to produce 3D polyethlylene gycol diacrylate (PEGDA) and GelMA scaffolds with precise internal geometries in a log pile or hexagonal pattern, that supported neuron and neurosphere growth, although large neurospheres were excluded from the scaffold [144].

Extremely high-resolution 3D patterning can be achieved using multi-photon polymerization systems, where a beam of ultrafast infrared laser is focused to a point within a volume of photopolymerizable material. Since the polymerisation reaction requires multiple photons, the resin only hardens in a very small volume near the focal point [151]. Multi-photon polymerisation has been utilized to print complex shapes such as microtowers or seashells for neural engineering [152,153]. Melissinaki et al. (2011) fabricated 3D polylactide-based (PLA) scaffolds via this technique; neural cells adhered to PLA filaments; however neurite outgrowth was not guided by PLA filaments on crosshatched structures [153]. The high precision of multiphoton polymerisation allows for the fabrication of complex forms such as 3D seashell models. PC12 cells migrated aligning themselves with the 3D seashell geometry, neurite extensions were projected between 3D objects with network interconnectivity [153]. The convoluted geometry of the seashell models appeared to promote neurite projection more so than the linear crosshatched structure; possibly because in vivo neural morphology is not orthogonal in nature.

#### 4.4.2. Extrusion Printed Scaffolds

Extrusion based 3D printing uses a robotically controlled syringe to extrude materials in a filamentous strand; which is written on the fabrication stage in a layer-by-layer approach. Extrusion printing requires biomaterials to have sufficient rheological properties, such as a high enough viscosity, to maintain the strand shape prior to crosslinking. Extrusion printing typically has lower resolution than inkjet or laser systems [154,155].

For neural applications, extrusion printing has been used to create 3D scaffolds from a range of materials including fibrin, where the aligned microstructure of 200 µm diameter fibrin fibers provided durotactic cues for Schwann cell alignment mimicking the bands of Bünger that Schwann cells form following nerve injury to guide axon regeneration. [156]. In another example, researchers extrusion printed 3D scaffolds from a poly 2-hydroxyethyl methacrylate (pHEMA) hydrogel with varying rod spacing, finding that a 40 µm spacing was optimal for cell penetration and neurite network formation [143]. Neural growth was restricted to the top layers in scaffolds with rods 30 µm apart, while larger spacings resulted in cells settling towards the bottom of the scaffolds [143].

The printability requirements for extrusion bioprinting typically favour materials which are far stiffer than the brain. However, some strategies have managed to side-step this issue, creating very soft 3D structures using extrusion printing. For example, Tan et al. (2017) used a cryogenic printing approach to create cuboid lattices with a compressive modulus of 490 Pa, matching well with stiffness of brain tissue [157]. PVA and Phytagel were extruded and froze on contact with a steel plate maintained below freezing temperature and upon completion of printing the temperature was raised, melting the ice leaving an intact 3D hydrogel suspended in water [158]. This method is yet to be applied to neural tissue engineering.

#### 4.4.3. Convergent Biofabrication

Recently, photopatterning techniques have been combined with other methods in convergent biofabrication strategies. For example, Sundararaghavan et al. (2010) combined photomask crosslinking with electrospun HAMA to introduce vascular like channels within the microporous 3D fibrous scaffolds; allowing mesenchymal stem cells (MSC) to penetrate throughout the 3D scaffold [159] (poor infiltration of cells is a recognised drawback of electrospun scaffolds) [140,159]). Lee et al. (2017) combined electrospinning and stereolithographic techniques by embedding aligned electrospun PCL or PCL and gelatin composite fibres in 3D printed crosshatched PEGDA hydrogel scaffolds [160]. The integrins within gelatin, combined with the topographical cues from the electrospun fibres promoted and guided neurite formation compared to the hydrogels without electrospun fibres, despite the stiffer environment [160].

Inkjet printing has also been used to decorate 3D scaffolds created by other techniques, for example to generate spatially controlled growth factor gradients on 3D hydrogel scaffolds to steer stem cell differentiation [161]. Patterned cell “tracks” of electrically conductive particles have been inkjet printed to guide cell and neurite growth [162].

#### 4.4.4. 3D Scaffold-Based Disease Models

A traumatic brain injury (TBI) model was created by dropping a weight from varying heights on 3D silk scaffolds with compartmentalized architectures filled with collagen and encapsulated primary cortical cells [130]. At 1 min post injury glutamate levels were comparable to the response of a rat TBI model, in vivo glutamate levels return to preinjury level in 5 min; the reuptake of glutamate by astrocytes was not recapitulated in this neuron monoculture [130].

Photopatterning 3D GelMA microgel rings has been developed as a way to model autapse formation; axonal circles developed in singly captured neurons [150]. Autapsic ‘positive feedback’ self-stimulation of excitatory neurons are thought to play a role neural hypersynchrony which may lead to seizures [108,163].

## 5. 3D Bioprinting

Interactions among multiple cell types determines the structure and function of all tissues. The brain is very complex with many regions and sub regions, for example it is estimated that there are 500–1000 different gray matter regions [164,165]. The major cell types within brain tissue include neurons, oligodendrocytes, astrocytes, microglia, and endothelial cells, of which there are multiple subtypes. There are thought to be 50–250 neuronal subtypes [164]. Cell populations and densities vary between regions in a spatially defined manner orchestrating the tissue structure. For example neocortical tissue consists of six layers: The *lamina molecularis* has relatively few neurons, the thin *lamina granularis externa* is densely populated with small neurons, the *lamina pyramidalis externa* has medium pyramidal neurons, the *lamina granularis interna* consists of small stellate neurons, the *lamina pyramidalis interna* has large pyramidal neurons, and the *lamina multiformis* has polymorphic neurons [166]. Exquisite spatial control of differing cell populations would be of great benefit for the establishment of in vitro neural models to mirror the spatial heterogeneity of brain tissue in vivo.

Microfluidic casting utilizing polydimethylsiloxoane (PDMS) chips has been utilized to recreate complex layered 3D constructs, the dynamic environment offered by perfusion of microfluidic chips is a major advantage [72,79,167]. However, due to the PDMS chip the method is restricted to layers in a horizontal configuration [168], only allowing bidirectional analysis of cell interactions. 3D printed scaffolds have given new insights in neural modelling and have potential therapeutic applications regarding nerve conduits; but are limited by the seeding process. Cell penetration throughout a scaffold limits cell patterning and cells often have 2D morphology along scaffold surfaces [143]. Bioprinting refers to applications where cells are formulated within in a printable material called a bioink. Bioprinting overcomes the limitations of cell seeding techniques; it allows specific controlled placement of the cell laden bioink in a reproducible manner, allowing simultaneous spatial control of cells and materials [169,170,171].

In 3D bioprinting cell laden hydrogels are typically deposited in liquid form in the desired shape prior to being crosslinked into a gel within minutes [172]. Prior to cell proliferation the bioink serves as a rudimentary ECM [52,53,155,170,173]. Extrusion printing and inkjet printing are commonly employed methods of 3D bioprinting. Extrusion based methods typically utilize stiffer gels that are crosslinked as they are extruded in a string like manner, whereas inkjet-based and microvalve methods are more suitable for softer less viscous gels as they deposit drop by drop [174].

### 5.1. The Challenge of 3D Bioprinting of Soft Structures

The goal of 3D bioprinting is to mimic the native microenvironment of in vivo tissue. A major hurdle to 3D biofabrication of soft structures, such as the brain, is the shaping of soft hydrogels into 3D structures with high spatial resolution that are an anisotropically accurate mimic of the brain microstructure. A gel with brain like mechanical properties ~500 Pa will sag under its own weight due to gravity resulting in a loss of shape due to the extremely soft characteristic of the gel. Soft 3D scaffolds have been developed with cryogels or cryogenic printing, however cells were seeded on scaffolds after the exposure to subzero temperatures [133,134,135,158,175], which is known to affect cell viability studies [176,177,178].

A more straightforward approach is to use support structures, which support the soft structure during printing and during the maturation of cells to prevent deformation of the construct. This support structure needs to be biocompatible with the printed cells without affecting cell viability.

### 5.2. Support Structure Methods

Support structures can be classed into two broad categories: Permanent support structures or sacrificial scaffolds. A sacrificial support involves the printing of a structure, a support matrix, which can easily be removed after printing, often through a washing step [172]. Therefore, sacrificial scaffolds support the permanent material as it cures or matures to preserve the 3D printed shape. Metals, ceramics, and polymers are often used as scaffold substrates. However, the removal or degradation of these constructs can harm cell viability or result in toxic breakdown products [124,172].

#### 5.2.1. Sacrificial Support Scaffolds

The materials used for sacrificial supports vary greatly, however removal without harming cells is essential. Sacrificial scaffolds that dissolve quickly or require vigorous washing/flushing for removal are only appropriate for stiffer more cohesive gels, softer gels tend to disintegrate in the washing step [179]. Water soluble carbohydrate glass has been extrusion printed for the biofabrication of vascular junctions [180], melt electrospun sucrose microfibers have been utilized as sacrificial scaffolds in the biofabrication of interconnected channels for bone defect repair [82].

Hinton et al. (2015) developed a method of 3D printing soft materials called freeform reversible embedding of suspended hydrogels (FRESH), using a thermoreversible sacrificial support bath; unlike most 3D printing techniques this method is not restricted to layer-by-layer fabrication as demonstrated by the printing of a continuous 3D helix [148]. The support bath consisted of a 4.5% gelatin microparticle slurry, which acts as a rigid body under low shear stress and as a viscous liquid at high shear stress; meaning there was little mechanical resistance as the printing nozzle moved through the support bath, yet the extruded hydrogel was held in place. Crosslinking reagents were mixed into the gelatin slurry, upon completion of printing the gelatin was melted off at 37 °C and replaced with culture media leaving the crosslinked 3D hydrogel. Collagen, fibrin, Matrigel, and alginate hydrogels were printed at 22 °C in complex structures, including hollow chambers with a resolution of 200 µm. An alginate printed dog bone had an elastic modulus of 102 ± 27 kPa [148]. A concern using this technique with bioprinting is that the gelatin microparticles are melted off at the same temperature used for cell growth, so it is limited to fast crosslinking methods. Another study adapted this method using a sacrificial bath of alginate microparticles and culture media supplemented with xanthan-gum which was removed via enzymatic action. The support bath was gradually aspirated and replaced with media containing alginate lyase, and used to bioprint a simplified, mini heart model incorporating living cells [147]. FRESH has yet to be applied to neural cultures. The gentle nature of the technique suggests it may be suitable for neuron printing, although 3D deposition of extremely soft materials (<100 Pa) is yet to be demonstrated.

#### 5.2.2. Indirectly 3D Bioprinting

Indirect bioprinting refers to the printing of complex support structures and infusing the support structure with cells encapsulated in hydrogel [124,173]. Naghieh et al. (2019) indirectly 3D bioprinted primary rat Schwann cells in varying concentrations of alginate, using a sacrificial gelatin scaffold [181]. A 20 layered gelatin sacrificial scaffold was 3D printed and then impregnated with a cell-laden alginate hydrogel. The alginate was crosslinked through CaCl_2_ addition, before the sacrificial gelatin template was melted away at 37 °C. However, the softest hydrogel, 0.5% alginate, had a modulus of 2.5 kPa; and degraded within 3 h [181]. Cell viability was increased due to the presence of interconnected pores created via indirect 3D printing of sacrificial scaffolds [181].

### 5.3. 3D Bioprinting of Neurons

#### 5.3.1. Extrusion Based 3D Bioprinting of Neural Structures

Extrusion printing is commonly employed in bioprinting due to its economy, ease of use, and capability to print with high cell density with a wide range of materials [154,170]. Resolution is dependent upon nozzle diameter typically in the range of 50–500 µm [155].

Parameters such as nozzle diameter, dispensing pressure, and material viscosity during bioprinting submit cells to the stresses of shear force, tension, and compression; if excessive these forces can result in deformation and cell damage. Shear stress can be minimized by using lower pressure/propelling force, less viscous materials, and larger diameter nozzles [155,170]. Cell viability after printing is therefore often a principal focus of bioprinting studies; functionality is less often assessed. 

**Figure 4:**
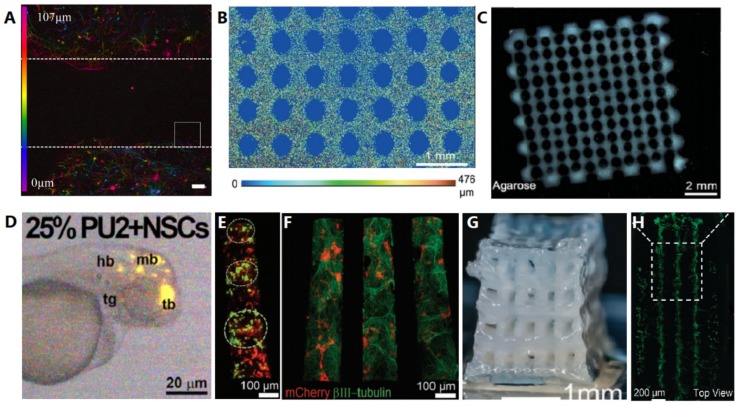
Extrusion bioprinted neural and iPSC derived cultures: (**A**) Layered cortical neurons in top and bottom layer RGD-GG [186]; (**B**) iPSCs in Al-Ag-CMC [183]. (**A**,**B**) Colour coded *z* axis. (**C**) Al-Ag-CMC printed grid [182]. (**D**) Zebrafish injected with bioprinted fluorescently labelled neural progenitor cells (NPCs) in 25% polyurethane (PU), fb = forebrain, mb = midbrain, hb+hindbrain and tg = trigeminal ganglion [184]. (**E**) Bioprinted alternating oligodendrocyte progenitor cells (OPCs) (red) and NPCs (green) in silicon channel 24 h post print, image adapted from [185] and (**F**) after four days in culture. (**G**) 3D printed alginate structure, (**H**) bioprinted NPCs in channels during assembly of 3D printed alginate structure, top view [185]. Images reproduced with permission from [182,183,184,185,186], scale bars as shown.

Lozano et al. (2015) printed discrete layers of polysaccharide gellan gum-RGD (peptide modified biopolymer) and primary rat cortical neurons. The gellan gum-RGD was crosslinked via CaCl_2_ or 5x Dulbecco’s Modified Eagle Medium (DMEM) and three layers were printed with neurons encapsulated in the bottom and top layer. Axon extensions spread throughout layers penetrating the acellular middle layer; neurons did not migrate between layers and networks formed within layers only (Figure 4A). Axon/dendrite extension was slightly improved using DMEM compared to CaCl_2_, although cells were more clustered compared to non-printed cast moulded 3D cultures.

Gu et al. (2016) extrusion printed human iPSCs that were differentiated post printing into neurons, astrocytes and oligodendrocytes, creating a neural mini-tissue 3D lattice (refer to Figure 4C). The bioink was a composite of alginate, agarose, and carboxymethyl-chitosan (Al-Ag-CMC) crosslinked post printing via CaCl_2_ immersion. Printed constructs had a compression modulus of 4.73 kPa which decreased to 0.8 kPa at 10 days post printing where it stabilized, closer to the stiffness of native brain [64]. Neurites with interconnections were detected but were limited perhaps due to the 3D environment being too stiff for optimal neurite outgrowth [182]. Synaptogenesis and functional GABAergic neurons developed after 24 days. In a later study this technique was used to show that iPSCs develop into embryoid bodies of three germ lineages when cultured in non-specific cell culture media [183] (Figure 4B). Differentiation into neural lineages involved culturing cells in media with specialized media supplemented with neurotrophins to induce an intermediate progenitor phase followed by a neural maturation phase. Mature neural cells were detected at 30 days post printing and bicuculline induced synchronous calcium spiking of two cells indicated functional neurons with possible network functionality at 40 days post printing [183]. This methodology of bioprinting iPSCs has potential for use in creating patient specific disease models.

Hsieh et al (2015) used fused deposition extrusion printing to bioprint thermoresponsive biodegradable polyurethane dispersions of PCL diol & poly (D, L-lactide) diol (PU) with encapsulated murine NSCs; encapsulated 3D constructs were implanted into zebrafish that had sustained brain injuries [184] (Figure 4D). Remarkably NSC laden bioink of 25% PU was found support NSC proliferation and to restore function to brain injured zebrafish; coiling rate was restored to 87% of uninjured control and hatching rate was restored to 67% of uninjured control. Bioinks composed of PCL diol & poly (D, L-lactide) diol with concentrations <25% were found to be too soft to maintain a 3D form [184]. A notable study conducted by Joung et al. (2018) developed a bioengineered spinal cord combining bioprinting with 3D printed scaffolds in the only example of functional neurons with extensive axon propagation from bioprinted NPCs. Spinal NPCs (sNPCs) and oligodendrocyte progenitor cells (OPCs) bioprinted in precise alternating points within 3D printed single channels, refer to Figure 4E,F and during the fabrication of 3D printed layered scaffolds [185] (Figure 4G,H). Both sNPCs and OPCs were differentiated from iPSCs prior to printing; OPCs were included for spinal cord regeneration applications as oligodendrocytes support axonal regeneration and myelinate demyelinated axons. Silicone was printed in single channels of 150 µm diameter and cured for 5 h, then Matrigel laden bioinks laden with OPCs or NPCs were dispensed separately at 4 °C in alternating points with a distribution resolution of 200 µm. After four days β- III tubulin positive axons spread throughout the channel. After 14 days NPCs were found to have differentiated into mature glutamate responsive neurons with functional network formation; inferred from the simultaneous calcium flux recordings of 10 cells throughout a channel with synchronous responses to K+ and glutamate [185]. Joung et al. (2018) then used an alginate and methylcellulose (Ag/MC) blend to 3D print a layered 1.5 × 5 mm scaffold with 3 × 3 150 µm wide channels while either the NPC or OPC laden bioink was simultaneously printed in the scaffold. The scaffold was crosslinked via CaCl_2_ immersion, cells were viable with some axonal elongation in all layers three days post printing. Axon propagation of NPCs in the coculture with OPCs bioprinted in the single silicon channel were increased compared to monoculture NPCs within the multilayered Ag/MC scaffold; whether due to presence of OPCs, the increased culture time of one day, or lack of exposure to crosslinking CaCl_2_ is unclear. Although this is a spinal cord scaffold the principles of bioprinting during support scaffold assembly could be applied to CNS neural tissue engineering.

#### 5.3.2. Inkjet Bioprinting of Neurons

**Figure 5:**
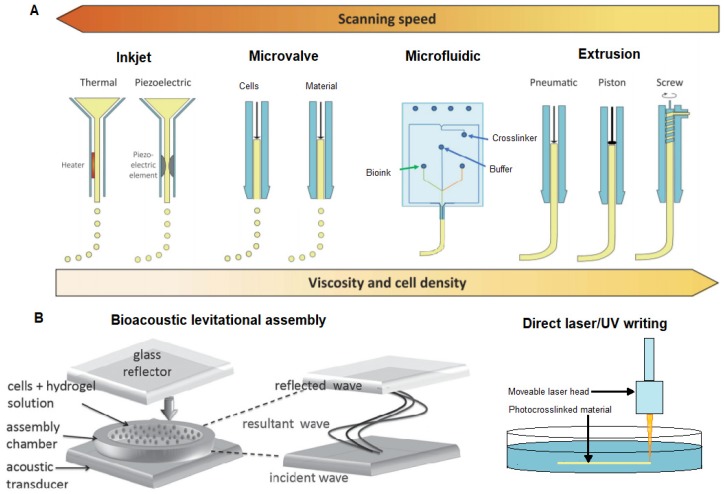
Methods of bioprinting neurons. (**A**) Nozzle-based methods: Inkjet and microvalve printing deposit drops on demand and are more appropriate for soft viscous materials. Microfluidic printing also uses soft viscous materials but deposits a filament as crosslinking occurs prior to deposition. Extrusion printing deposits a continuous filament and requires higher viscosity materials, image adapted from [174,196]. (**B**) Nozzle free methods include bioacoustic levitational assembly and UV laser writing [193]. Images reproduced with permission from [174,193,196].

Although extrusion is popular in 3D bioprinting it is only suitable for viscous gels, whereas inkjet deposits drop by drop making it more appropriate for soft gels [187] (Figure 5A). In inkjet printing, the jetting process is typically controlled by rapidly forcing a droplet of liquid out of a small orifice. This jetting can be produced either by thermal or piezoelectric control.

Inkjet printing allows higher print resolution with precise cell placement due to the droplets deposited being in the picolitre range, the challenge with inkjet printing is building layers in the z direction due to the low viscosity of relevant materials, the only 3D inkjet printed neural model consisted of stacked layers of 2D patterned gels [118].

**Figure 6:**
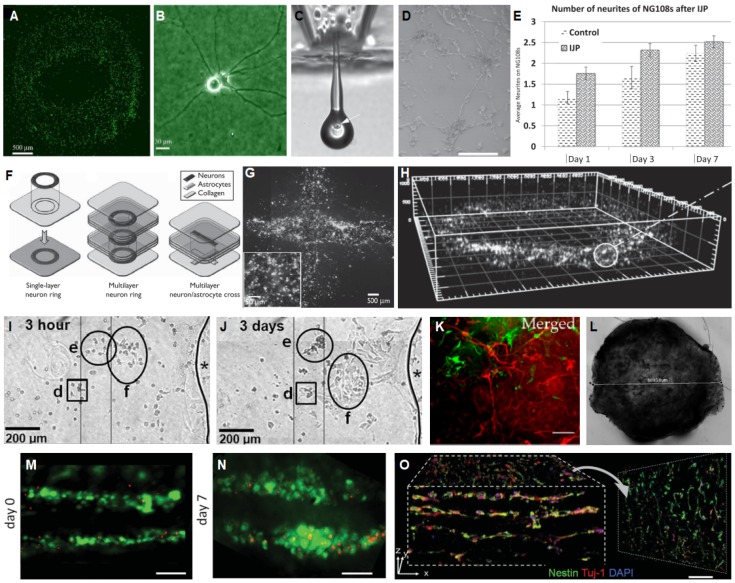
Inkjet and other bioprinting techniques: (**A**) Thermally inkjet printed rat hippocampal neurons onto collagen sheet [189], scale bar 500 µm. (**B**) A rat cortical neuron 13 days after being thermally inkjet printed [189], scale bar 30 µm. (**C**) Close up image with arrow showing neuron in piezoelectric induced jet [192]. (**D**) Printed neurons at seven days after piezoelectric printing [191], scale bar 100 µm. (**E**) Piezoelectric printed neurons had initially higher neurite formation compared to non-printed controls, which stabilized by day seven [191]. (**F**) Schematic explaining the rationale used to microvalve print hippocampal neurons and astrocytes in (**G**,**H**), scale bar in (**G**) 500 µm, inset 50 µm and (**H**) each grid is 500 µm by 500 µm [194]. Neurons migrate and differentiate toward vascular endothelial growth factor (VEGF) releasing fibrin gels marked by * in (**I**,**J**) [195], scale bar 200 µm. (**K**) Microfluidic printed NPCs differentiate into glial (GFAP+ green) and neural (β-Tubulin III+ red) lineages, scale bar 50 µm [196]. (**L**) Microfluidic bioprinted cylinder with diameter of 6693.0 µm [196]. Live (green) and dead NPCs (red) at (**M**) day zero and (**N**) seven days after bioacoustic levitation (BAL), scale bar 250 µm [193]. (**O**) One week after BAL NPCs differentiated into neurons in 3D layers (β-Tubulin III+ red), progenitor cells (Nestin+ green) [193]. Images reproduced with permission from [189,191,192,193,194,195,196].

In a pioneering work, Xu et al. (2005) utilized a commercial desktop printer equipped with thermal print heads to print embryonic motor neurons onto a 2D sheet of collagen, forming a neural ring after two days; and 40 µm dendritic extensions were observed in two cells after seven days [188]. In a later study, rat hippocampal neurons were thermal inkjet printed onto 2D fibrogen gels. Cells were exposed to temperatures of approximately 300 °C and shear stress of 10 m s^−1^ for 5 µs, which may account for the cell viability being only 72.4%, although it should be noted that no non-printed control viability was reported [189] (Figure 6A). Five layers of thermally inkjet printed fibrin sheets were stacked to form a 3D structure with a softness of 2.92 MPa, cell survival over 15 days was improved in 3D versus 2D culture [189] (Figure 6B). Electrophysiological behaviour of 2D thermally inkjet printed neurons were comparable to non-printed controls via patch clamp recording [189]. In contrast to these results Kador et al. (2019) found that the current required to stimulate action potentials in thermal inkjet printed retinal ganglion cells (RGC) had to be increased by a third over non-printed controls to elicit the same response [190]; similar to the electrophysiological properties of axomized neurons in brain injury models (Greer et al., 2012). Kador et al. (2016) developed a 3D scaffold with PLA electrospun fibres to guide axon outgrowth in an inkjet printed retina model. Embedding the scaffold in Matrigel incubated with CaCl_2_, cells were printed in an alginate bioink, which crosslinked the cells embedded within the hydrogel scaffold. Neurites were classified as axons if they were long with a consistent diameter or as dendrites if the diameter was thicker at cell soma with tapered extension. Neurite outgrowth was aligned with the PLA printed pattern in 71.9% ± 8.2% of axons and 49.3% ± 4.0% of dendrites after three days [190], however this was reduced compared to 81% axonal alignment observed on scaffolds simply seeded with cells; which had the same electrophysiological properties as 2D non printed controls [137]. The conditions cells are exposed to in thermal inkjet printing may be too extreme to apply for the formation of functional neural networks, essential to the development of disease models of epilepsy; especially because directed axon propagation was not improved by seeding techniques.

Varying voltage applied during piezoelectric printing was demonstrated to not affect cell viability or metabolic activity in a 2D model [191] (Figure 6D). For voltages ranging from 70 V to 240 V printed mouse neuronal NG108-15 cells, human fibroblasts and porcine Schwann cells; had cell viability of 86%–96%, 82%–92%, and 89%–92%, respectively; compared to >90% in non-printed controls [191]. Neural cells could be printed for 40 min before significant loss of cell number due to sedimentation in the printhead, when pre-flushed with trypsin. By day seven neurite length of printed cells was on average 65 µm, significantly longer than 55 µm of non-printed controls [191] (Figure 6E). Lorber et al. (2013) did not find that piezoelectric printing advanced neurite length, however length increased by ~190 µm when RGCs were printed over a layer of printed glial cells demonstrating that glial cells retain their growth promoting characteristics after piezoelectric inkjet printing [192]. Cell viability was not affected by piezoelectric printing, the cell population, however, was reduced in printed samples compared to non-printed controls, by 57% and 33% for glial and RGC, respectively. Imaging during printing of the printhead nozzle where cells experience the most shear stress showed that cells were not distorted during printing (Figure 6C). Post jetting imaging showed that a large number of cells had adhered to the glass capillary walls near the nozzle, suggesting that the reduced cell number was due to cell sedimentation, as opposed to cell destruction, during the piezoelectric printing process [192]. Whether piezoelectric printing affects the functionality of neurons has not been elucidated and should be an object of future study.

#### 5.3.3. Other Methods of 3D Printing Neural Cells

Bioacoustic levitation (BAL) is a nozzle free method were cells are levitated by the reflection and interference of standing acoustic waves; crosslinking methods are required to be rapid to entrap levitated cells before cell settling occurs [193] (Figure 5B). 3D fibrin gels with multilayered NPCs produced via BAL had an elastic modulus of 474 Pa, mimicking brain tissue [64,193]. Over 30 days the neural progenitor cells differentiated into immature neurons, with some neurite extension.Although by this timepoint neurons had migrated from the distinct layers that were visible at day eight and some cell settling had occurred towards the bottom of the 3D gel [193] (Figure 6M–O). Perhaps if BAL was combined with other methods such as electrospinning the neural migration could be controlled.

Microvalve printing is a drop by drop or continuous filament method where droplet formation, with volumes in the order of 10 nanoliters, is controlled via adjustments to the applied pressure and valve opening duration (Figure 5A). Printing resolution is determined by the inter-dispensing distance and drop volume. Lee et al. (2009) used a stepwise layering approach to bioprint neurons and astrocytes in 3D collagen. Sodium bicarbonate (NaHCO_3_) was alternated with dispensed collagen layers and bioprinted cells, completing crosslinking and entrapping cells in their spatially defined position. For cell patterning at least one acellular collagen layer separated cell laden layers; distinct cell layers required separation by multiple acellular layers (Figure 6F). 3D multilayered neurons were bioprinted in a ring pattern with interconnecting neurites and co-cultures of rat neurons and astrocytes in a cross (Figure 6G,H); demonstrating that microvalve printing allows patterning of different cell types in the one 3D construct [194]. This same group used microvalve printing to fabricate a layered 3D NPCs laden collagen construct with a thrombin crosslinked fibrin gel printed adjacently. The fibrin gel incorporated soluble vascular endothelial growth factor (VEGF) which was released gradually over three days [195]. NPCs migrated towards the chemoattractant VEGF-releasing fibrin gel (Figure 6I,J); increased proliferation and markedly increased branched morphology with neurite projections was observed in the presence of both VEGF and fibrin, in contrast to either alone [195]. These were the first 3D bioprinted collagen neural cultures, given the promising results of functional neurons and neural network formation in 3D bulk collagen models [20,58,118] further development and electrophysiological assessment of this method is warranted. This method of localising growth factors could be combined with 3D bioprinting to recapitulate the morphogen gradients that drive organogenesis in vivo [155]. Application of this method with droplets in the picolitre range to increase the resolution could increase the precision of cell placement, perhaps via inkjet printing. 

Like microvalve printing, microfluidic printing relies on pressure to control the flow rate; however, the bioink is crosslinked prior to deposition at a triton shaped junction where the crosslinking channel, buffer channel, and bioink channel meet (Figure 5B). Crosslinking takes place rapidly and the crosslinked fibres are ejected prior to deposition, cells are protected from shear stress due to the formation of a protective sheath around cells as crosslinking occurs from both sides [196]. De la Vega et al. (2018) used a microfluidic bioprinting method to print NPC cells with a resolution of 176 µm in a 3D cylindrical fibrin scaffold for spinal cord repair [196]. Extensive neurite extension and network formation were visible at 30 days (Figure 6K), by then the printed structure was degraded, yet the cells maintained the 3D shape demonstrating that this method promotes cells to excrete their own ECM [196] (Figure 6L).

UV laser writing (Figure 5B) was used to 3D bioprint mouse NPCs in GelMA or a nano bioink consisting of electrically conductive graphene nanoplatelets combined with GelMA (G-GelMA) [197]. NPCs differentiated into mature neurons with limited neurite extension, likely due to the stiffness of the printed gel, which was ~30 kPa (Gel-MA only). Neurite length was slightly improved in the G-GelMA compared to GelMA alone, possibly due to the conductive properties of the graphene or perhaps the addition of transparency reducing graphene resulted in a softer gel with less photo crosslinking [197]. The conductivity and effect of electrical stimulation on bioprinted NPCs should be explored further. 

#### 5.3.4. 3D Bioprinting Summary

Piezoelectric inkjet bioprinting has given the highest resolution in printing neural cultures 25 ± 5 µm [191] with high cell viability; however cells were simply printed into wells of a culture plate. Cell sedimentation in the printhead reduces the number of printed cells [192]; which can be reduced to increase print time by flushing the printhead and tubing with trypsin [191]. Interestingly the number of neurites formed was initially increased in printed samples perhaps due to the piezoelectric effect stimulating neural differentiation [87,191]. The electrophysiological behaviour of neurons after piezoelectric printing has not been explored; thermally inject printed neurons have been observed to have similar electrophysiological properties to damaged neurons [190,198], although other studies have found no difference compared to non-printed cells [118] (Table 2). Insofar inkjet printing has been used to pattern neurons in 2D [118,188,190,191,192], the only 3D construct was five layers of fibrin hydrogel sheets patterned with rat hippocampal and cortical neurons [118]. The low viscosity of bioinks used in inkjet printing impedes construction in the z direction, which could possibly be overcome by printing within a support structure [124]. Microvalve printing is also a droplet based technique using larger droplet volumes and has been used to create 3D collagen based constructs with 3D neural network formation within discrete layers, co-culture cell patterning and incorporated growth factor impregnated regions [194,195,199]. Microvalve printing has been demonstrated to be amenable to printing 3D constructs with different hydrogel compositions in different regions; VEGF releasing fibrin crosslinked via enzymatic action of thrombin, and pH-based crosslinking of collagen. Neural migration towards the VEGF illustrated that spatially controlled chemoattractant regions incorporated into 3D designs can be used to further control neural placement and subsequent behaviour via bioprinting [195]. Although 3D, these were not free-standing structures and the spatial control was limited to simple patterns within layers. Microfluidic bioprinting where the crosslinking occurs prior to droplet deposition resulting in continuous filament deposition, is more akin to extrusion printing than other droplet based techniques [196]. Microfluidic bioprinted fibrin 3D cylinders supported NPCs differentiation into neurons and astrocytes with neurite extension and network formation. After 30 days cellular ECM production was sufficient to maintain the 3D shape even after the scaffold degraded.

The most spatially defined 3D neural models have been fabricated via extrusion printing, typically as lattice structures [182,183,184,200]. Insofar extrusion bioprinting of neural cells in 3D has been limited to bioprinting the one cell type throughout the construct [182,183,184,200] (Table 2). Gu et al. differentiated iPSCs into neurons, astrocytes and oligodendrocytes; however, the cell types were not spatially defined within the structure [182]. Joung et al. (2018) patterned bioprinted OPCs and NPCs within 3D printed channel support structures, although there was extensive axon propagation within the channel this was really 2D patterning within a 3D printed structure. The mechanical characteristics of the bioink itself were not defined, the excellent neurite extension and functional network formation suggest the bioink was very viscous; hence why a support structure was used [185]. If a sacrificial scaffold was used in place of a permanent scaffold perhaps networks could form between channels over time. Gu et al. (2017) developed the longest living 3D bioprinted neural construct at 40 days. Neurite extension was somewhat limited because of the stiffness of the mechanical environment [182]; however synaptogenesis did occur and functionally mature neurons were established. Two neurons showed synchronous calcium spiking behaviour which suggests they responded in tandem; however to demonstrate network functionality multiple synchronous responses are required [58,104]. The only 3D bioprinted neural constructs with demonstrated functionality have been extrusion printed [182,183,184,185]; although functionality was inferred from functional recovery in a zebrafish model or calcium flux assays (Table 2). Electrophysiological assessments are yet to confirm functional neurons and neural networks of 3D bioprinted constructs. Extrusion based methods of 3D bioprinting are limited to viscous self-supporting bioinks, which do not mimic the mechanical properties of the native brain. However, by employing support structures or cryogenic methods this limitation could be overcome.

## 6. Conclusions and Outlook

Patient specific disease modelling of neural disorders is feasible with iPSCs directed differentiation into neural tissue. Creation of a neural disease model that accurately mimics brain tissue is a difficult task due to the highly complex tissue microarchitecture of the brain. Brain tissue is heterogeneous regarding cell populations and mechanical properties, and very soft. Brain organoids have been developed but results have had poor reproducibility, limiting their application in disease modelling. Organoids are limited in size and develop a necrotic core if grown too large due to lack of vascularization.

3D neural models require biomaterials that mimic the mechanical properties of the brain and support the development of functional neural networks. Hydrogels mimic the poro-viscoelastic properties of brain tissue and have been demonstrated to support neural growth. Functionally active neural networks in 3D have been established in bulk gels of low concentration, and therefore soft, hydrogels of crosslinked collagen, hyaluronic acid and alginate. Bulk gels are simplistic due to the random cell distribution and cell settling effects, the small pore size also limits mass transport throughout the gel restricting the size of the model. 3D scaffolds introduce macropores to facilitate mass transport, 3D printed scaffolds have specifically designed pores or channels, in a highly reproducible automated manner. Cells are seeded onto scaffolds, and often have poor infiltration of the scaffold. Cells grow along the surfaces of the scaffold resulting in unnatural constraints affecting morphology and network disconnection between scaffold areas. 3D bioprinting of cells within the scaffold structure is a way of overcoming these limitations, while also allowing precise cell patterning. 

3D bioprinting of neural models has been restricted by the constraints of developing a bioink that is soft enough to allow functional network formation and synaptogenesis, yet printable and capable of maintaining a 3D shape. These conflicting requirements present technical challenges, which may be overcome by using sacrificial support scaffolding, sacrificial baths or novel crosslinking methods. Most neural bioprinting studies have focused on cell survival post printing, developing bioinks and demonstrating neural differentiation in printed constructs. Demonstrating established functional networks in bioprinted 3D in a reproducible manner is the first milestone to be achieved. The notion of using bioprinting to specifically arrange cells to replicate heterogeneous cell distribution within the brain has only been explored minimally with prototype models of alternating or criss-cross cell patterns, and only with two different cell types. Patterning of multiple cell types including different glial populations with neural cells is necessary to mirror the varying cell populations within different brain regions. In the context of disease modelling, iPSCs could be used to grow patient specific organoids that spontaneously develop distinct brain regions. Cells from each brain region could be harvested and encapsulated in a suitable bioink for patterning via 3D bioprinting in a reproducible manner. An alternative method would be to incorporate differentiation cues within the bioink to develop spatially directed differentiation in a bioprinted organoid approach. Durotactic and haptotactic cues combined with growth factor gradients could further guide cell migration and neurite extension to achieve complexity in designed tissue microarchitecture.

Vascularized neural models have not yet been developed. Incorporation of channels to allow mass transport of waste, nutrients, and oxygen will allow the survival of larger neural models. Dynamic flow as used in microfluidic devices will further recapitulate the in vivo microenviroment, in place of vasculature. For disease modelling replication of the blood brain barrier is important to determine whether prospective treatments will reach the target tissue at an effective concentration, if they are effective at all.

3D bioprinting of neural models is still in the early stages of development and offers great potential for exquisite spatial bioink patterning to recapitulate the microarchitecture of brain tissue. Although 3D bioprinted neural disease models have not yet been developed, the potential advantages over animal models include species and patient specific disease modelling. First, neural models must be bioprinted with demonstrated functionally active neural networks; only then can bioprinted neural disease models be compared to animal disease models. The technology to create 3D models of brain tissue by bioprinting neurons is still in its infancy, but holds great promise as a new tool for studying the brain.

## Figures and Tables

**Table 1:** Summary of biomaterials used to culture neurons in 3D.

Biomaterial	Conc. (*w*/*v*)	Mechanical Properties	Cell Type	Culture Details	Network Formation?	Functional?	Ref
**Agarose**	0.5%	NA	Day-18 rat cortical neurons	14 days	No	NA	[84]
**Agarose**	1.0%	NA	DRGs	6 days	No	NA	[110]
**Alginate**	0.25%	180 Pa(G’)	Rat hippocampal NSC	7 days	No	NA	[115]
**Alginate + PLGA microspheres**	1.0%	NA	Rat hippocampal NSC	8 days	No	NA	[116]
**Alginate**	0.1%	10 Pa (G’)	Day-17 rat cortical neurons	4 weeks	Yes	Yes ^1,2^, network multiple sites calcium flux-network	[112]
**CMC**	0.5%	104 ± 13.46 Pa(G’)	Rat hippocampal cortical neurons, DRGs and NSCs	14 days	No	NA	[111]
**Collagen type I**	0.04%	NA	Day-18 rat cortical neurons	10 days	Yes	NA	[84]
**Collagen** **type I**	0.3%	NA	NPCs	30 days	No	Yes individual neurons only ^1^	[93]
**Collagen type I**	0.2%	57–377 Pa (G’)	Chick dorsal root ganglions	5 days	NA	NA	[117]
**Collagen type I**	0.04%	10 Pa (G*)	Rat dorsal root ganglions	24 h	NA	NA	[95]
**Collagen type I**	0.04%	NA	Day 13 rat cortical and subcortical neurons	24 days	Yes	Yes, neuron whole cell patch clamp	[20]
**Collagen type I**	0.04%	NA	Embryonic day-18 rat hippocampal neurons	35 days	Yes	Yes, networkmultiple sites connected MEA	[58]
**Collagen type I**	0.1%	NA	Embryonic day-18 rat hippocampal neurons	21 days	Yes	Yes, neuron whole cell patch clamp	[118]
**HA-SH + peptides**	4–6%	188 ± 42 Pa (G’)	H9 human embryonic stem cells	10 weeks	No	No, patch clamp recording cells immature	[100]
**HA-SH+RGD**	3%	400 Pa (G’)	Embryonic mouse hippocampal NPC	21 days	NA	NA	[99]
**HAMA (+peptides)**	1%	130 Pa (E)	NPCs from normal and Rett syndrome patient derived hiPSCs	3 weeks	Yes	Yes, neuron whole cell patch clamp	[40]
**HAMA**	0.5%	200 Pa (E_c_)	mixed glial cells	14 days	No	NA	[13]
**HAMA**	0.5%	510–1410 P (E_c_)	NPCs from normal and Down syndrome patient derived hiPSCs	28 days	No	NA	[103]
**HAMA**	1.5%	3000 Pa (E_c_)	Rat ventral midbrain NPCs	14 days	No	NA	[102]
**HA-TG/Lys-Gln**	0.5%	100 Pa (G’)	Embryonic rat cortical neurons	2 months	Yes	Yes ^1^, network multiple sites calcium flux-network	[104]
**GelMA/HAMA**	(3.5/0.5%)	1100 Pa (E_c_)	E12.5 hindbrain cell	15 days	Yes	NA	[109]
**Matrigel**	0.05%	NA	Rat embryonic cortical neurons and astrocytes	60 days	NA	Yes, neuron whole cell patch clamp	[61]
**GelMA**	4% and 8%	NA	PC12 cells	7 days	NA	Maybe, healed mouse brain injury	[107]
**GelMA**	3%	680 Pa(G’)	iPSC derived NPC	7 days	NA	Maybe, restored functional recovery after SCI	[108]
**Fibrin**	0.4–1%	400–1200 Pa (G’)	Mouse embryo derived NPSC	14 days	Yes	NA	[114]
**PEG +RGD+ MMP degrading peptides**	4.4%	NA	iPSC derived NPC, EC and microglial cells	21 days	NA	NA	[18]

Notes: NA: Not assessed. G’ = storage modulus; G* = complex dynamic modulus, E = Elastic (Young’s) modulus; E_c_ = compressive modulus; ^1^ Functionality is inferred in calcium flux assay; ^2^ without challenging the cells with signal blockers it is unconfirmed that the calcium spiking activity was from neural electrical activity alone.

**Table 2:** Overview of different methods used to print neural cells.

Print Type	Cell Type	Bioink	Cell Morphology	Cell Viability	Mechanical Properties	Functionally Active	Ref
Extrusion	Primary rat cortical neurons	gellan gum-RGD	Dendrite extension, interconnecting networks.	74 ± 2%	NA	NA	[186]
Extrusion	Murine NPCs	PCL diol & poly (D, L-lactide) diol	Globular, no dendrite extension	proliferation	680 Pa (*E*)	Yes (zebra fish brain injury model) ^1^	[184]
Extrusion	Human NPCs	alginate, agarose, carboxymethyl-chitosan.	Limited dendrite extension	90% (day 7)	7.5 kPa (*E_c_*), 4.75 kPa (*E*)	Yes, neuron, calcium flux assay ^2^	[182]
Extrusion	iPSCs	“	“	Not reported	“	Yes, calcium flux assay ^2^	[183]
Extrusion ^3^	Schwann cells	Composite of alginate, RGD and YIGSR	Globular	95% (Day 0), 95% (Day 7)	40–14 kPa (day 0–15) (*E*)	NA	[145]
Extrusion ^3^	Schwann cells	Composite of fibrin, HA and Factor XIII	Bipolar, aligned with strands	98%	NA	NA	[201]
Extrusion	Schwann, neuronal (rodent) glioma (human)	Pluronic F-127, gelatin, HA	Globular	metabolic activity inc.	6.7 kPa (*G’*)	NA	[200]
Extrusion	sNPC and OPCs	50% Matrigel	Axon propagation	>75% (day 4)	~55 kPa (*E*)	Yes, calcium flux assay ^2^	[185]
Thermal inkjet	rat neurons	Liquid media	Dendrite extension	72.4%	NA	Yes, neuron, patch clamp	[189]
Thermal inkjet	Rat neurons	Liquid media	NA	NA	2.92 MPa (substrate) (*E*)	NA	[189]
Thermal Inkjet	Rat retinal ganglion cells	Liquid media containing BDNF & CNGF	Neurite extension	NS w.r.t. controls	NA	Yes, patch clamp	[190]
Peizo inkjet	Rat glial, retinal ganglion cells	2D Liquid media (DMEM)	Dendrite extension	69 ± 5% 69 ± 12%	NA	NA	[192]
Peizo inkjet	NG108-15,Human fibroblasts, Porcine Schwann cells	2D Liquid media (DMEM) +10% feotal calf serum	Neurite extension	86–96%, 82–92%, 89–92%	NA	NA	[191]
Bio-acoustic levitation	Neural progenitors (from human ESC)	Fibrin gel	Some neurite extension	‘majority’	474 Pa (*G’*)	NA	[193]
Microvalve	Rat embryonic neurons	Layered collagen (0.2%)	NA	78.6%	NA	NA	[199]
Microvalve	Embryonic rat neurons and astrocytes	Layered collagen (0.112%)	Neurite extension, 3D network	78.6%, 78.7%	NA	NA	[194]
Microvalve	Murine neural stem cells	Layered collagen (0.116%), integrated VEGF- fibrin gel	Neurite extension	92.89 ± 2.32%	NA	NA	[195]
Microfludic	iPSC derived NPCs	Fibrin, chitosan, alginate	Neurite extension, 3D networks	>81%	NA	NA	[196]
Direct laser writing	Chick spinal cord cells	Media	some neurite extension	Not quantified	NA	NA	[202]
Direct laser writing	hiPSCs	15% hyaluronic acid in media	NA	82 ± 1%	NA	NA	[203]
UV laser writing	Mouse NPCs	10% GelMA, 10% GelMA + graphene nano platelets	neurite extension	Not quantified	30 kPa (GelMA) (*E_C_*)	NA	[197]

NA: Not assessed; *E_C_* = compressive modulus; *E* = Elastic modulus; G’ = storage modulus. ^1^ Coiling rate restored to 87% of uninjured control, hatching rate restored to 67% of uninjured control. ^2^ Functionality is inferred in calcium flux assay. ^3^ Technically a seeded scaffold, however scaffold was bioprinted with Schwann cells.
